# Relationship between BMI and affective disorders: results from a multicenter observational study

**DOI:** 10.1192/j.eurpsy.2025.384

**Published:** 2025-08-26

**Authors:** C. Toni, B. Della Rocca, M. De Vincenzo, E. Mancuso, V. Dello Stritto, V. Coppola, G. Sampogna, M. Luciano, A. Fiorillo

**Affiliations:** 1 Psychiatry, University of Campania “Luigi Vanvitelli”, Naples, Italy

## Abstract

**Introduction:**

Patients suffering from severe mental disorders have a reduced life expectancy of approximately 10-25 years compared to the general population. This mortality gap is mainly due to physical comorbidities among which metabolic disorders play a significant role.

**Objectives:**

In our study we used the Body Mass Index (BMI), an indicator of general health that can be easily calculated in daily clinical practice, to investigate how weight and the different psychopathological and psychosocial dimensions mutually influence each other in patients with mental disorders.

**Methods:**

This naturalistic observational multicenter study was carried-out in 7 Italian university centers (Universities of Campania “L. Vanvitelli,” Catania, Magna Graecia of Catanzaro, Cattolica del Sacro Cuore of Rome, Padova, Sapienza University of Rome, and Tor Vergata of Rome). Patients were recruited if they: 1) had diagnosis of bipolar disorder (BD) or major depressive disorder (MDD) according to DSM-5 criteria; 2) had an age between 18 and 65 years; 4) were in a stable phase of the disease (total score < 9 on the Hamilton Rating Scale for Depression and a score of ≤11 on the Young Mania Rating Scale). Affective temperaments were assessed with the Munster Temperament Evaluation of the Memphis, Pisa, Paris, and San Diego, impulsivity with the Barratt Impulsiveness Scale, and suicidal ideation with the Columbia Suicide Severity Rating Scale.

**Results:**

A total of 598 patients were recruited, of which 60.9% affected by DB and 39.1% by MDD. Univariate analyzes revealed an association between higher BMI and male gender (p<0.001), BD diagnosis (p<0.001), high levels of impulsivity (p<0.05), presence of psychotic symptoms during the acute phases of illness (p<0.05), greater number of hospitalizations (p<0.01), cigarette smoking (p<0.05) and depressive temperament (p<0.001). Furthermore, patients treated with lithium (p<0.05), antiepileptics (p<0.05) and first-generation antipsychotics (p<0.001) had a significantly higher BMI compared to those not taking the aforementioned pharmacological treatments.

**Conclusions:**

The results of our study highlight a strong link between BMI and some clinical outcomes in patients with affective disorders. The routinary assessment of these outcomes would be useful for the early identification of potential metabolic comorbidities as well as to identify patients at higher risk to develop a worse outcome.

**Disclosure of Interest:**

None Declared

